# Endovascular Recanalization of Non-Acute Vertebrobasilar Occlusion in Progressive Posterior Circulation Ischemia: A Case Report

**DOI:** 10.3390/neurolint18100183

**Published:** 2026-09-25

**Authors:** Gamaliel Wibowo Soetanto, Elvan Wiyarta

**Affiliations:** 1 Department of Neurology, St Borromeus Hospital, Bandung 40132, Indonesia; 2 Intensive Care Unit, University of Indonesia Hospital, Depok 16424, Indonesia; elvan.wiyarta@ui.ac.id

**Keywords:** vertebrobasilar, posterior circulation, non-acute occlusion, recanalization, stenting, ischemia

## Abstract

Background: Unlike acute basilar artery occlusion, non-acute vertebrobasilar occlusion has limited evidence for intervention and requires individualized risk assessment. Case Presentation: A 62-year-old woman developed progressive vertigo, diplopia, dysarthria, and gait ataxia over ten days. Dual antiplatelet therapy was started after posterior circulation ischemia was suspected, but symptoms continued to progress. MRI showed acute to subacute left cerebellar infarction, and CTA showed absent antegrade basilar opacification with collateral distal filling. DSA showed distal left vertebral artery occlusion, retrograde basilar filling through posterior communicating artery collaterals, and hypoplastic right vertebral artery. Endovascular recanalization was performed after multidisciplinary review and informed consent. Intraluminal crossing, stepwise angioplasty, and stent reconstruction restored antegrade basilar flow without angiographic complications. NIHSS improved from 5 to 2 at discharge, and mRS was 1 at 30 days. No follow-up vascular imaging was available. Conclusions: This case illustrates technical feasibility in selected non-acute vertebrobasilar occlusion, but does not establish medical treatment failure, causal recovery, generalizable safety, or long-term durability.

## 1. Introduction

Posterior circulation stroke is a distinct cerebrovascular syndrome affecting the vertebrobasilar system that supplies the brainstem, cerebellum, and occipital lobes, and it accounts for a significant share of ischemic strokes with high severity when large vessels are involved [1]. Registry data show that posterior circulation events often result from embolic and large-artery disease, with a significant hemodynamic component in patients with occlusive vertebrobasilar lesions, a critical concern given the brainstem’s limited tolerance for reduced blood flow [1]. Basilar and vertebrobasilar occlusive disease has a poor natural history with medical therapy alone, especially when collateral flow is insufficient or exhausted, resulting in recurrent or progressive ischemic symptoms [2,3].

Evidence from acute basilar artery occlusion cannot be directly extrapolated to non-acute vertebrobasilar occlusion, where occlusion age, atherosclerotic plaque, perforator anatomy, lesion length, and restenosis risk create a different problem. Current evidence for chronic or non-acute intracranial vertebral and vertebrobasilar recanalization remains limited to small observational series and case report [4,5,6]. These data support only exploratory use in highly selected patients.

Symptomatic chronic total occlusion of the intracranial vertebral or vertebrobasilar segment remains underrepresented in randomized trials and is often managed medically due to perceived procedural complexity and risks of perforator injury, dissection, and restenosis [4,5]. Historically, surgical and endovascular approaches to ischemic stroke have evolved substantially, although high-level evidence for non-acute vertebrobasilar recanalization remains lacking. Prior observational series and case reports have already demonstrated the technical feasibility of endovascular recanalization for symptomatic non-acute vertebrobasilar occlusion [4,5,6]. Accordingly, this report does not propose a new patient selection algorithm or a novel recanalization technique. Its contribution is limited to a detailed technical illustration of intraluminal crossing, stepwise angioplasty, and long-segment stent reconstruction in a non-acute vertebrobasilar occlusion with collateral-dependent distal filling and hypoplastic contralateral vertebral inflow, together with explicit discussion of the uncertainties surrounding medical treatment failure, physiological selection, and long-term durability. The case is reported in accordance with the SCARE guideline [7].

## 2. Case Illustration

A 62-year-old Asian female with a medical history notable for long-standing hypertension, type 2 diabetes mellitus, dyslipidaemia, and active tobacco use was admitted to our comprehensive stroke centre with progressive ischemic symptoms attributable to posterior circulation compromise. The clinical presentation evolved over approximately ten days and was characterized by persistent vertigo, nausea, gait instability, binocular diplopia, and fluctuating dysarthria. Symptoms initially exhibited a waxing and waning pattern but demonstrated progressive deterioration. There was no history of acute loss of consciousness, seizure activity, or focal limb weakness. No stroke or TIA had been documented before the current 10-day illness. Dual antiplatelet therapy with aspirin 80 mg once daily and clopidogrel 75 mg once daily was started after posterior circulation ischemia was suspected. Reported adherence was based on patient and family confirmation. Symptoms continued to progress during this short-course regimen, which was not considered definitive failure of optimized medical therapy.

On neurological examination at admission, the patient was alert and oriented. Cranial nerve assessment revealed gaze-evoked nystagmus and mild dysarthria. Extraocular movements were intact, without ophthalmoplegia. Limb motor strength was preserved bilaterally, and no pyramidal signs were present. Marked truncal and gait ataxia were observed, consistent with cerebellar involvement. Sensory examination was unremarkable. The National Institutes of Health Stroke Scale score was 5, driven by cerebellar signs and speech disturbance. Although NIHSS was mild, the patient had progressive posterior circulation symptoms, including persistent gait ataxia, diplopia, and dysarthria. The decision was based on clinical progression and collateral-dependent basilar perfusion rather than NIHSS severity alone. Vital signs were stable except for elevated blood pressure measuring 176 over 98 mmHg. Laboratory evaluation including complete blood count, coagulation profile, renal function, and inflammatory markers showed no significant abnormalities.

Baseline brain magnetic resonance imaging demonstrated structural and diffusion abnormalities within the posterior fossa. Axial T2-weighted imaging revealed parenchymal signal abnormality involving the left cerebellar hemisphere, consistent with subacute ischemic injury within the posterior inferior cerebellar artery territory (Figure 1B). Diffusion-weighted imaging confirmed restricted diffusion in the same region, supporting an acute-to-subacute ischemic process without evidence of haemorrhagic transformation or mass effect (Figure 1C). To further evaluate the underlying vascular pathology, computed tomography angiography of the head and neck was performed. Three-dimensional reconstruction demonstrated absence of antegrade opacification of the basilar artery with preserved distal posterior circulation supplied via posterior communicating arteries (Figure 1A).

After MRI and CTA had shown posterior circulation infarction with vertebrobasilar occlusive disease, diagnostic cerebral angiography was performed to define the vascular anatomy and assess recanalization feasibility. Digital subtraction angiography of the dominant left vertebral artery demonstrated complete occlusion at the distal V4 segment with abrupt termination of contrast and no antegrade basilar artery opacification (Figure 2A). In the absence of prior vascular imaging establishing the duration of occlusion, the lesion was classified clinically as non-acute rather than as a definite chronic total occlusion. In the delayed angiographic phase, the distal basilar artery and bilateral posterior cerebral arteries opacified retrogradely via posterior communicating artery collaterals, demonstrating collateral-dependent posterior circulation filling (Figure 2B). This angiographic pattern was considered anatomically concerning in the context of progressive symptoms, but it did not quantify tissue perfusion or cerebrovascular reserve. No CT perfusion, MR perfusion, SPECT, PET, or quantitative angiographic perfusion study was available. Therefore, hemodynamic failure could not be objectively established. The right vertebral artery was hypoplastic and did not provide effective basilar inflow, reinforcing a single vessel dependent posterior circulation with limited physiologic reserve. Recanalization was considered because of progressive posterior circulation symptoms, absence of large brainstem infarction, dominant left vertebral supply, hypoplastic contralateral inflow, distal basilar reconstitution, and crossable lesion morphology on DSA. Recanalization would have been avoided if there was no distal target, heavy calcification, unsafe wire trajectory, major brainstem infarction, or poor functional reserve. Calibrated occlusion length and formal stump morphology were not prospectively recorded. Because these parameters could not be reliably reconstructed from the available angiographic record, no retrospective occlusion length estimate or standardized stump classification was assigned. Their absence limits reproducibility of the crossing strategy and prevents morphology-based comparison with previously reported chronic occlusion series.

Following multidisciplinary cerebrovascular discussion, endovascular recanalization was considered as an individualized exploratory intervention because symptoms continued to progress during index hospitalization, in a setting of collateral-dependent basilar filling with no large established brainstem infarction. Importantly, the patient had received only a short course of dual antiplatelet therapy, which did not constitute an adequate trial of optimized medical management. Therefore, the intervention should not be interpreted as rescue therapy after established medical treatment failure. Continued medical therapy, procedural uncertainty, and the risks of stroke, perforation, hemorrhage, thrombosis, restenosis, reocclusion, and death were discussed with the patient and family. Informed consent was obtained before the procedure. Periprocedural hemorrhagic risk was assessed clinically and radiographically rather than with a formal hemorrhage prediction score. Relevant considerations included the extent of established infarction, absence of hemorrhagic transformation or significant mass effect on baseline MRI, absence of a large established brainstem infarction, baseline blood pressure, hematological and coagulation parameters, concomitant antiplatelet therapy, the requirement for intraprocedural anticoagulation, and procedure-specific risks of vessel perforation and reperfusion-related hemorrhage. The admission blood pressure was 176/98 mmHg, and strict blood pressure control was maintained during and after the intervention. No formal hemorrhage risk score was applied. Stopping criteria included unsafe wire trajectory, suspected subintimal passage, contrast extravasation, flow-limiting dissection, distal embolization, or unstable thrombus. Bail-out measures included repeat angiography, anticoagulation adjustment, balloon tamponade for perforation, thrombus aspiration or antithrombotic rescue, and strict blood pressure control. OA-PICA bypass was not planned as immediate rescue because this would not address acute basilar perforator injury, rupture, or thrombosis.

The procedure was performed under general anaesthesia in a biplane microangiography suite. General anaesthesia was selected for immobility, airway protection, controlled ventilation, and strict blood pressure control during prolonged posterior circulation recanalization. We acknowledge that this prevented real-time neurological monitoring. Right common femoral arterial access was obtained and systemic anticoagulation was initiated with intravenous unfractionated heparin, titrated to maintain an activated clotting time between 250 and 300 s, with serial checks and redosing as required. Proximal support was established using a Ballast guiding catheter (BALT, Montmorency, France). Distal access was achieved with a SOFIA 6 Fr 115 cm aspiration catheter (MicroVention, Aliso Viejo, CA, USA) advanced into the cervical segment of the left vertebral artery to optimize coaxial stability and reduce unsupported microcatheter length across the lesion. Under high-magnification roadmap guidance, the proximal cap was engaged with a Transend 300 0.014-inch microwire (Boston Scientific, Marlborough, MA, USA) shaped with a short atraumatic curve. The occlusion was traversed using controlled forward tension and microcatheter support, with intermittent contrast injections to confirm intraluminal progression. Successful intraluminal crossing was demonstrated angiographically by distal microwire position within the vertebrobasilar circulation confirming true lumen re-entry (Figure 2C).

After true lumen confirmation, predilation was performed using a Gateway balloon 3 mm by 15 mm (Boston Scientific, Marlborough, MA, USA) with stepwise low-pressure inflations to establish an initial luminal channel while minimizing intimal injury and perforator compromise risk. Residual stenosis and elastic recoil remained, consistent with intracranial atherosclerotic disease at the vertebrobasilar junction. Taken together with the patient’s vascular risk factor profile and the angiographic appearance of an established distal V4 occlusive lesion, these findings supported intracranial large-artery atherosclerotic disease as the presumed mechanism of the vertebrobasilar occlusion, rather than an isolated acute embolic event. Nevertheless, the angiographic findings alone cannot definitively exclude a concomitant cardioembolic mechanism, and the etiologic attribution should therefore be considered presumptive rather than definitive. Balloon angioplasty alone was considered insufficient because residual flow-limiting stenosis and elastic recoil persisted. Stenting was performed to maintain antegrade basilar flow, but the required length increased perforator coverage risk. Throughout the intervention, a stable coaxial system was maintained across the reconstructed segment, with the guiding catheter, distal access catheter, and microcatheter construct aligned to preserve support and minimize kickback during balloon inflation and device exchanges (Figure 2D). Definitive vessel reconstruction was performed with deployment of a LEO 5.5 × 75 mm braided self-expanding stent (BALT, France), spanning from the distal left vertebral artery across the vertebrobasilar junction into the basilar trunk. After angioplasty, residual flow-limiting stenosis and elastic recoil remained, and continuous scaffolding across the curved reconstructed segment was considered necessary. The LEO stent was selected by the treating operator because its length, deliverability, and conformability allowed continuous coverage of this trajectory. No calibrated vessel diameter measurements or quantitative tortuosity metrics were recorded; therefore, diameter mismatch or tortuosity cannot be presented retrospectively as formal device selection criteria. The device was used off-label for intracranial atherosclerotic occlusion. This operator-specific choice does not imply superiority over devices specifically intended for intracranial atherosclerotic stenosis, and regulatory labeling and device availability differ across jurisdictions. Potential disadvantages include lower radial force, thrombogenicity, incomplete apposition, thrombosis, and restenosis. The long stent was also recognized as off-label and risky because it could cover pontine and medullary perforators. Perforator assessment relied on high-magnification DSA, while vessel wall MRI was unavailable. Risk reduction included low-pressure angioplasty, avoidance of aggressive overdilation, and no further post-dilation after acceptable antegrade flow. Adjunctive aspiration and thrombus management was performed using Catch Max (BALT, Montmorency, France) to reduce intraprocedural thrombus burden and optimize luminal patency in the setting of non-acute occlusion recanalization.

Post-stent-deployment angiography demonstrated restoration of vessel caliber across the vertebrobasilar junction with improved luminal continuity and reduction in angiographic waist, indicating effective scaffold expansion against atherosclerotic plaque and recoil control (Figure 2E). Final angiography confirmed brisk antegrade flow through the basilar trunk and basilar apex with complete opacification of bilateral posterior cerebral arteries and no angiographic complications such as dissection, distal embolization, contrast extravasation, or visible perforator compromise (Figure 2F). Residual stenosis was estimated at less than 20 percent by visual assessment. Figure 2 shows baseline occlusion (A), delayed collateral filling (B), true-lumen crossing (C), access system configuration (D), immediate post-stent angiography (E), and final completion angiography (F).

Post procedurally, the patient was managed in the neurocritical care unit with strict blood pressure control and continued dual antiplatelet therapy. Follow up non-contrast head computed tomography showed no intracranial haemorrhage. Neurological examination improved progressively, with resolution of diplopia and significant improvement in gait stability. At discharge on day six, the National Institutes of Health Stroke Scale score had improved from 5 to 2. At 30-day clinical follow-up, the patient remained symptom-free and had an mRS score of 1. No follow-up CTA, MRA, or DSA was available, so stent patency, restenosis, and silent infarction could not be assessed. Accordingly, the term “technical success” in this report refers only to the immediate angiographic endpoint and should not be interpreted as evidence of durable stent patency, freedom from restenosis or reocclusion, or long-term clinical benefit.

## 3. Discussion

Progressive posterior circulation symptoms occurred despite angiographically patent collateral pathways in this case. The combination of subacute cerebellar infarction, fluctuating symptoms, and collateral-dependent basilar filling raised concern for impaired posterior circulation perfusion; however, collateral filling on DSA is an anatomical observation and does not establish physiological hemodynamic failure. Because no quantitative perfusion or cerebrovascular reserve study was performed, the severity and mechanism of flow compromise remain unproven. Accordingly, hemodynamic insufficiency in this report should be regarded as a clinical inference rather than a measured treatment selection criterion. The presence of subacute cerebellar infarction, fluctuating symptoms, and delayed collateral-dependent perfusion on angiography suggested ongoing flow insufficiency, a pattern that has been associated with recurrent ischemic events despite antithrombotic therapy in vertebrobasilar occlusive disease [8,9]. Although vertebrobasilar occlusive disease may be associated with recurrent or progressive ischemia, the present case does not establish that continued medical management would have failed or that revascularization was superior [8]. The intervention was therefore an individualized exploratory decision rather than an evidence-based rescue after an adequate trial of optimized medical therapy. In this case, intervention was individualized because symptoms progressed, basilar flow was collateral-dependent, contralateral vertebral inflow was hypoplastic, and no large brainstem infarction was present. Continued medical therapy was discussed as an alternative. These factors were weighed against risks of perforation, dissection, embolization, perforator infarction, hyperperfusion, thrombosis, restenosis, reocclusion, and death.

Because no prior vascular imaging confirmed occlusion for more than 30 days, this lesion is best described as a non-acute or possible acute-on-chronic vertebrobasilar occlusion rather than a definite CTO. From a technical standpoint, recanalization of non-acute vertebrobasilar occlusion differs fundamentally from acute thrombectomy and requires a strategy adapted to chronic atherosclerotic morphology. Intravascular crossing must prioritize true lumen traversal through fibrotic proximal caps while avoiding subintimal dissection or perforator compromise, which are major causes of procedural morbidity in intracranial CTO interventions [1]. In this case, the use of robust proximal support, a distal access catheter to shorten the unsupported segment, and controlled microwire advancement with frequent contrast confirmation was critical for maintaining intraluminal orientation, consistent with techniques described in prior CTO recanalization series [5].

Lesion preparation and reconstruction strategy are equally important determinants of outcome. Balloon angioplasty alone is frequently insufficient in intracranial atherosclerotic CTO due to elastic recoil and residual flow limiting stenosis, particularly at the vertebrobasilar junction where plaque burden and vessel curvature are pronounced [10]. In this setting, long-segment stent reconstruction using a braided self-expanding device provides sustained radial force and plaque scaffolding, reducing recoil and improving luminal continuity. However, this approach must be balanced against the risk of perforator coverage and in-stent restenosis, both of which remain unresolved concerns in intracranial stenting [1]. Final DSA without visible perforator compromise does not exclude microperforator ischemia. This risk was discussed before consent. This case should not be interpreted as evidence that long-segment basilar stenting is broadly safe. The absence of angiographic perforator compromise and the favorable early clinical course in this case suggest that careful sizing, precise deployment, and avoidance of aggressive overdilation may mitigate these risks.

Although immediate angiographic recanalization and early neurological improvement occurred in temporal sequence, this uncontrolled single case cannot establish that the neurological recovery was caused by stenting. The improvement in NIHSS from 5 to 2 and the mRS score of 1 at 30 days may also be explained by natural recovery from a relatively small subacute cerebellar infarction with supportive care and secondary prevention. We therefore report the clinical improvement descriptively and do not interpret it as evidence of treatment efficacy. The patient had also not completed optimized medical therapy; consequently, neither causal efficacy nor failure of medical management can be inferred from this case. This single uncontrolled case cannot prove causality. The patient had not completed optimized medical therapy. This case should not be interpreted as definitive failure of medical management. Follow-up was limited to 30-day clinical assessment without vascular imaging. Therefore, this report cannot determine stent patency, in-stent restenosis, reocclusion, or durability of the reconstructed vertebrobasilar segment. This limitation is particularly relevant given the use of a 75 mm braided stent spanning the vertebrobasilar junction. Longer-term vascular imaging, ideally at 6 to 12 months, would be required to evaluate durability. Quantitative perfusion imaging was not available. Hemodynamic compromise was inferred from the clinical course and collateral-dependent DSA filling rather than confirmed by CT perfusion, MR perfusion, SPECT, PET, or quantitative angiographic perfusion analysis. Long-term patency, restenosis rates, and durability of symptom relief following vertebrobasilar CTO stenting are not well defined, as existing evidence is limited to small observational cohorts with heterogeneous follow-up [1]. Moreover, patient selection remains critical, as recanalization may be futile or harmful in individuals with completed infarction, adequate collateral compensation, or limited functional reserve.

## 4. Conclusions

This case provides a descriptive technical example of immediate endovascular recanalization in a non-acute vertebrobasilar occlusion. It does not establish a new treatment indication, patient-selection algorithm, or evidence of clinical efficacy. Interpretation should remain cautious because optimized medical therapy was incomplete, hemodynamic failure was not quantified, and follow-up lacked vascular imaging. This procedure remains exploratory and requires careful selection, complication planning, and long-term imaging surveillance.

## Figures and Tables

**Figure 1:**
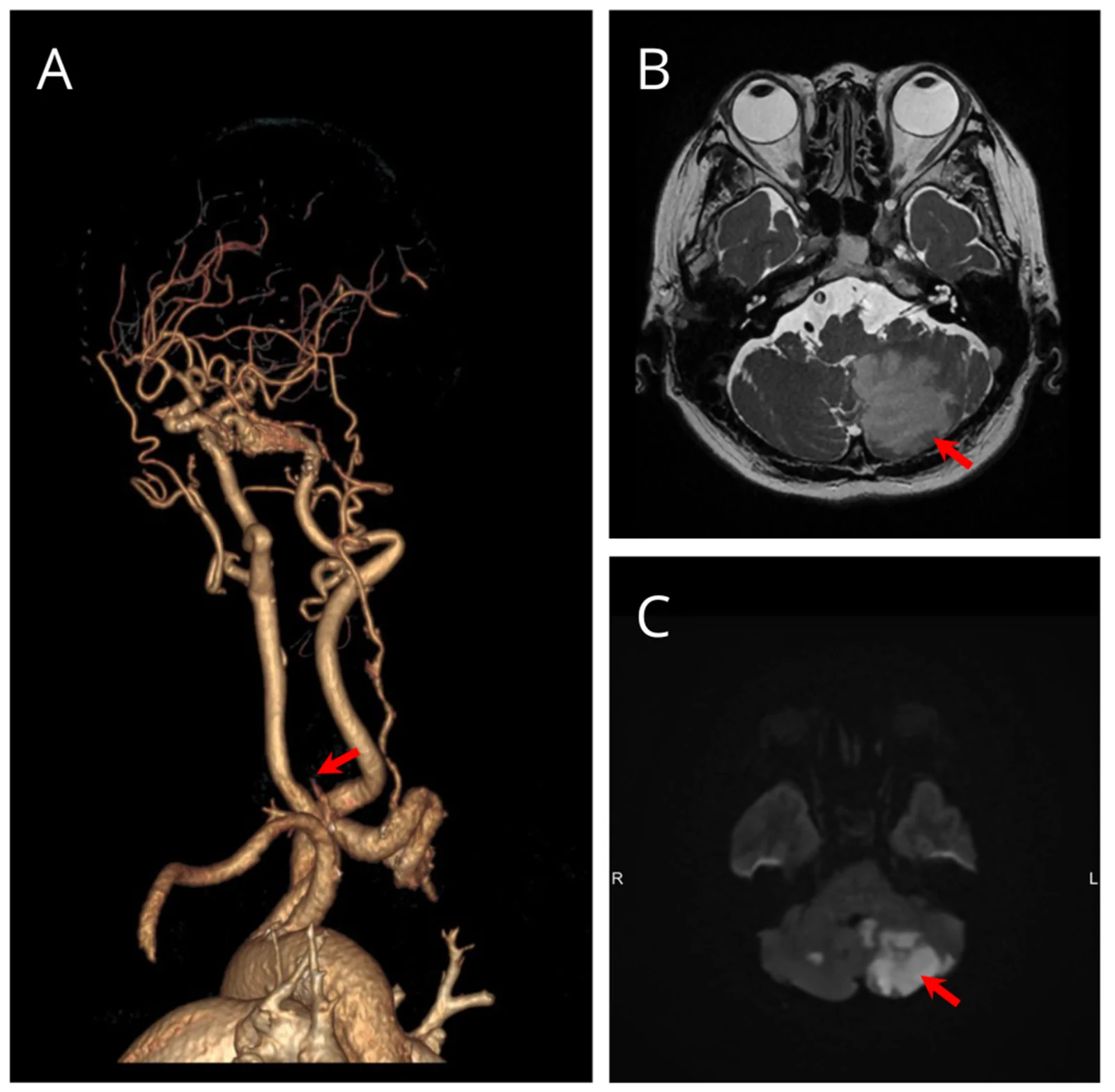
Baseline neuroimaging showing posterior circulation ischemia and vertebrobasilar occlusive disease. (**A**) Three-dimensional CT angiography demonstrates absent antegrade basilar artery opacification with distal posterior circulation filling through collateral pathways. (**B**) Axial T2-weighted MRI demonstrates signal abnormality in the left cerebellar hemisphere, consistent with subacute ischemic injury (arrow). (**C**) Diffusion-weighted imaging demonstrates restricted diffusion in the corresponding left cerebellar region, consistent with acute-to-subacute infarction (arrow).

**Figure 2:**
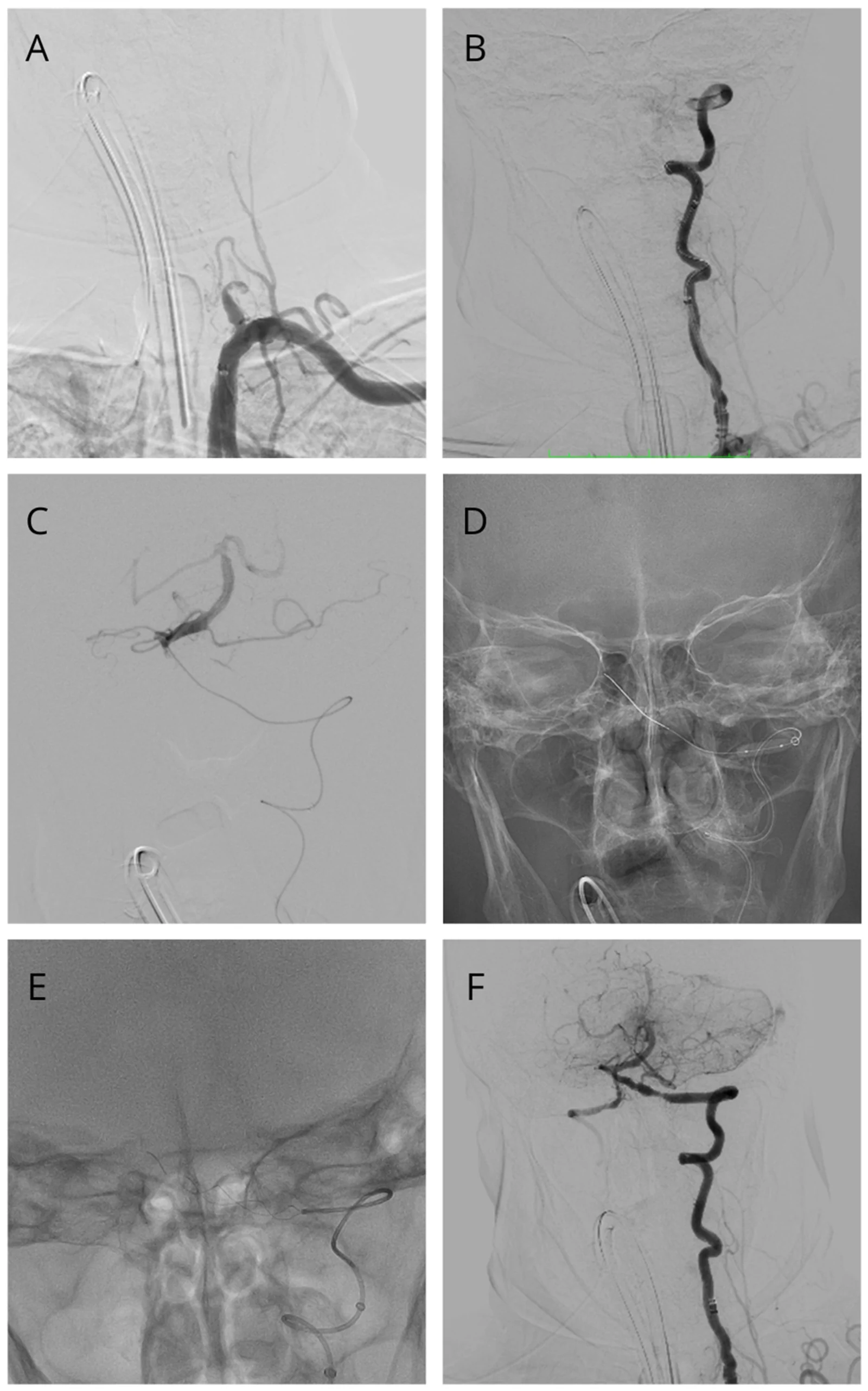
Digital subtraction angiography of the vertebrobasilar circulation before, during, and after endovascular recanalization. (**A**) Initial left vertebral artery injection demonstrates complete occlusion of the distal V4 segment with absent antegrade basilar flow. (**B**) Delayed phase demonstrates retrograde collateral filling of the distal basilar and posterior cerebral arteries. (**C**) Intraprocedural angiogram confirms successful crossing of the occlusion with microwire re-entry into the true lumen. (**D**) Working projection shows stable guiding, distal access, and microcatheter positioning across the reconstructed segment. (**E**) Post-stent angiogram demonstrates restored vessel caliber at the vertebrobasilar junction. (**F**) Final run confirms brisk antegrade basilar flow with complete posterior cerebral artery opacification and no complications.

## Data Availability

All data generated or analyzed during the study are included in this published article.
